# A case report of successfully treated nephrotic syndrome after renal angioplasty

**DOI:** 10.1186/s12882-019-1503-y

**Published:** 2019-08-06

**Authors:** Hee Jung Park, Ha Nee Jang, Hyun Seop Cho, Se-Ho Chang, Hyun-Jung Kim

**Affiliations:** 1 Division of Nephrology, Department of Internal Medicine, Gyeongsang National University Hospital, Jinju, South Korea; 2 Department of Internal Medicine, College of Medicine, Gyeongsang National University, Jinju, South Korea; 3 Institute of Health Sciences, Gyeongsang National University, Jinju, South Korea

**Keywords:** Renal angioplasty, Focal segmental glomerulosclerosis, Nephrotic syndrome, Renal artery stenosis

## Abstract

**Background:**

The standard treatment of renovascular hypertension accompanying renal artery stenosis (RAS) consists of angioplasty and administration of antihypertensive medication. Although nephrotic syndrome (NS) has been reported to be associated with RAS, the development of NS after revascularization of RAS is extremely rare.

**Case presentation:**

A 48-year-old man presented with uncontrolled hypertension and azotemia. The right kidney was atrophic, and RAS of the left kidney was suspected based on a post-captopril DTPA scan. His blood pressure stabilized after renal angioplasty; however, he complained of edema after 1 week. NS developed and was diagnosed as focal segmental glomerulosclerosis (FSGS) based on renal biopsy. He received an angiotensin receptor blocker. Proteinuria resolved after 1 year.

**Conclusions:**

FSGS rarely develops after angioplasty of renal artery stenosis. This is the first report of successful treatment of this condition using an angiotensin receptor blocker during 1-year follow-up.

## Background

Renal artery stenosis (RAS) is defined as a narrowing of one or both renal arteries or their branches and is a cause of renovascular hypertension [1]. Proteinuria can occur in patients with RAS, decreasing after angioplasty [2–4]. Nephrotic-range proteinuria in patients with RAS is uncommon, with most of these patients presenting with focal segmental glomerulosclerosis (FSGS) [5–9], defined as segmental glomerular scars involving some but not all glomeruli. Clinically, FSGS most frequently manifests as proteinuria and is present in up to one-third of adults with nephrotic syndrome (NS) [10]. However, nephrotic-range proteinuria has also been reported after angioplasty for RAS [11, 12], with FSGS occurring in the ipsilateral kidney after angioplasty. This report describes a patient who developed FSGS after renal angioplasty of a single functional kidney with RAS. This is the first report of successful treatment of this condition using an angiotensin receptor blocker (ARB) during 1-year follow-up.

## Case presentation

A 48-year-old man was referred to our hospital for evaluation of azotemia and uncontrolled blood pressure. Ten years earlier, he had been diagnosed with an intracerebral hemorrhage and underwent stereotactic hematoma aspiration. Due to uncontrolled hypertension at that time, he was evaluated for secondary hypertension at admission; however, no specific findings were observed on a captopril renal scan. Since then, he had been prescribed antihypertensive drugs by a private clinic.

At his first visit to our hospital for uncontrolled hypertension, his blood pressure was 160/90 mmHg despite triple therapy with amlodipine, carvedilol, and hydrochlorothiazide. His blood urea nitrogen concentration was 23.7 mg/dl (reference, 6.0–20.0 mg/dl), his serum creatinine (sCr) concentration was 1.45 mg/dl (reference, 0.6–1.2 mg/dl), and his estimated glomerular filtration rate (eGFR) using the CKD-EPI equation was 57 ml/min/1.73 m^2^. His serum total protein concentration was 7.4 g/dl, his serum albumin concentration was 4.5 g/dl (reference, 3.5–5.2 g/dl), his total cholesterol concentration was 292 mg/dl (reference, 120–220 mg/dl), his low-density lipoprotein-cholesterol concentration was 195 mg/dl (reference, 0–130 mg/dl), his triglyceride concentration was 73 mg/dl (reference, 0–200 mg/dl), his plasma renin activity (PRA) was 19.32 ng/ml/hr. (reference: supine, 0.32–1.84 ng/ml/hr.; upright, 0.60–4.18 ng/ml/hr), and his spot urine protein-to-Cr ratio (uPCR) was 0.4 g/g without hematuria.

**Fig. 1:**
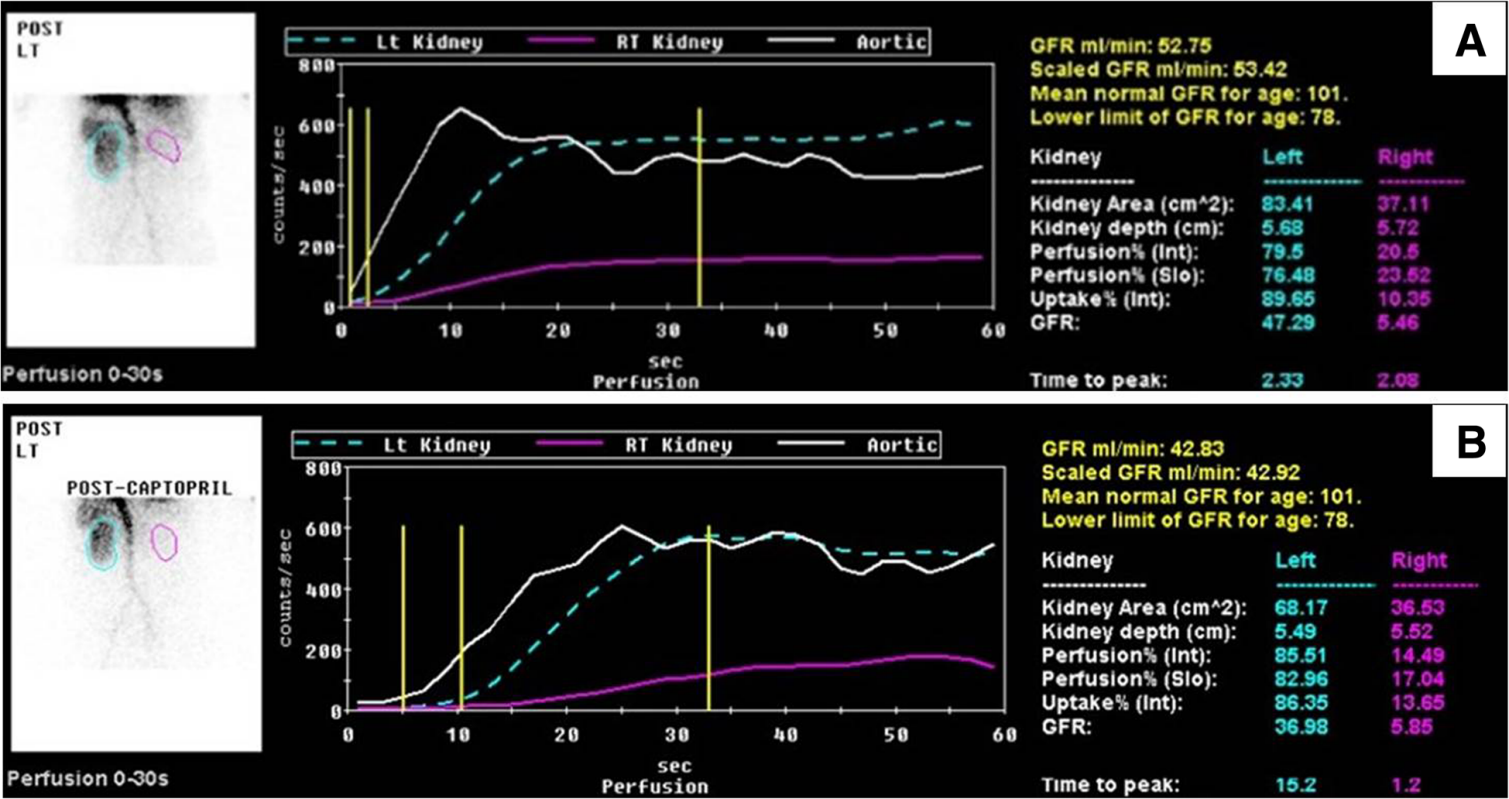
**a** Basal and **b** post-captopril DTPA renograms. A comparison showed that the eGFR was reduced > 10% in the left kidney, whereas the right kidney was non-functional

**Fig. 2:**
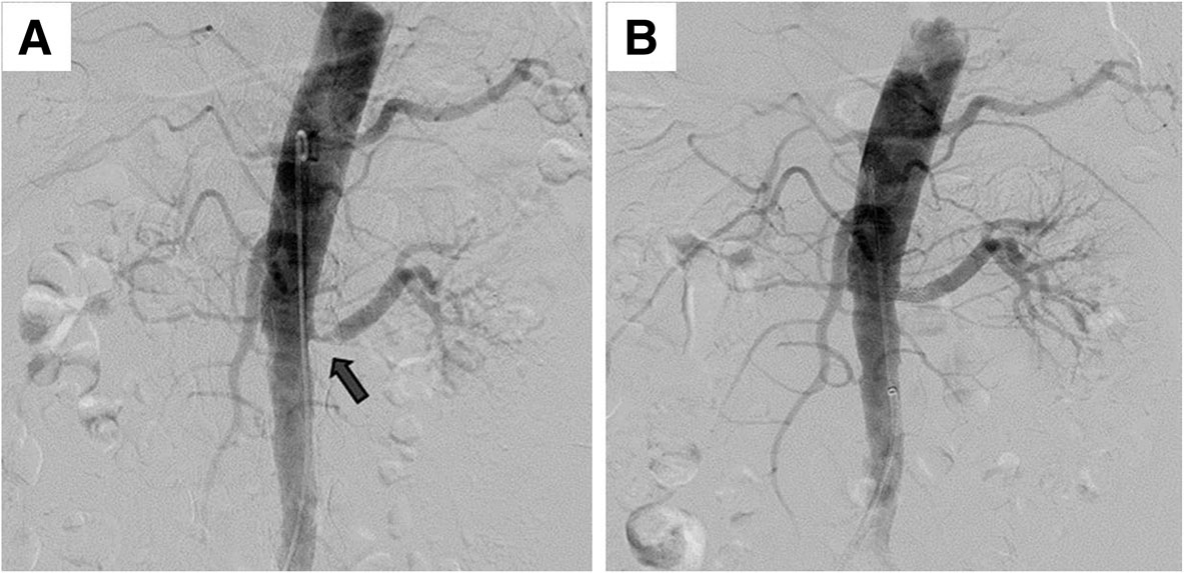
**a** Renal artery CT angiogram showing severe stenosis of the left renal artery at its site of origin (80%, arrows). **b** Successful insertion of a stent into the left renal artery

**Fig. 3:**
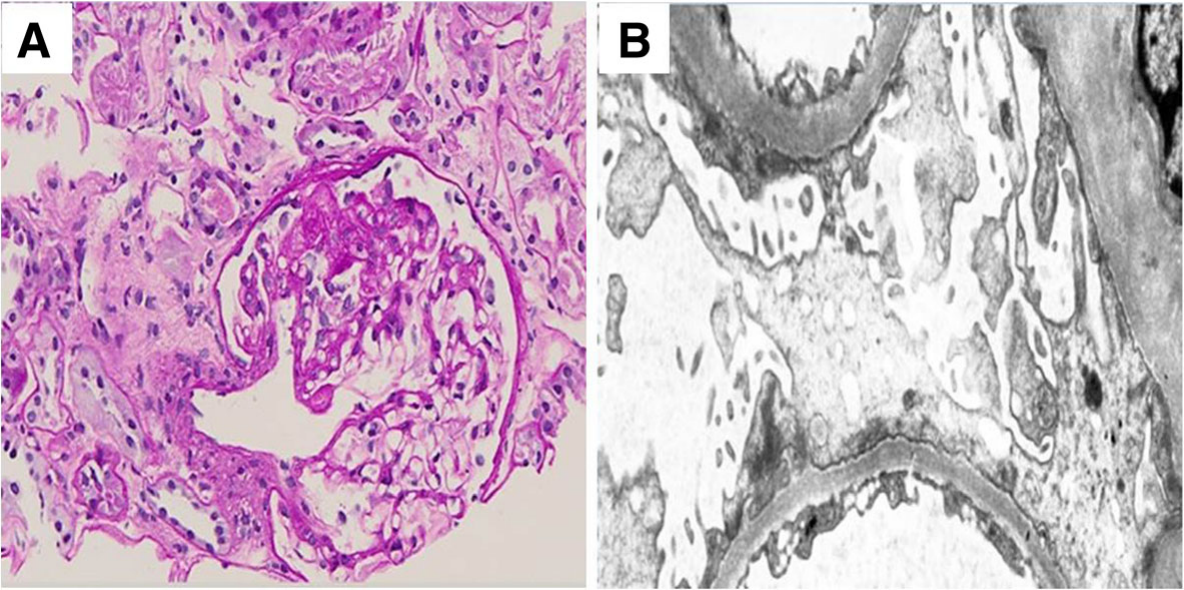
**a** Biopsy of the left kidney showing a glomerulus with segmental sclerosis (PAS, × 200). **b** Electron photomicrograph showing focal areas of mild epithelial foot process fusion; electron-dense deposits were not observed

A renal ultrasonogram revealed that his right and left kidneys measured 6.3 and 10.3 cm, respectively. Compared with a basal scan, a post-captopril DTPA renogram showed that the eGFR was > 10% lower in his left kidney, whereas his right kidney was non-functional (Fig. 1). A renal arteriogram showed 80% stenosis of his left renal artery, into which a stent was successfully inserted (Fig. 2), whereas his right kidney was faintly visible through collaterals. Four weeks after the procedure, the patient had developed marked generalized pitting edema compatible with NS, as shown by a uPCR of 11.6 g/g, and dyslipidemia. His PRA was 60.60 mg/dl/hr. at this time. Serological evaluation showed that he was negative for antibodies against immunoglobulins, complement, and hepatitis B and C viruses, as well as for antinuclear antibody. A biopsy of his left kidney resulted in a diagnosis of FSGS (Fig. 3). The patient was prescribed daily doses of the ARB candesartan, which reduced his uPCR to 2.2 mg/mg and his sCr to 1.30 mg/dl after 9 weeks. After 1 year, he recovered to his previous proteinuria range, and renal Doppler ultrasonography showed that his left renal artery was intact.

## Discussion and conclusions

Nephrotic-range proteinuria (> 3.5 g/day) is rare, but can occur in patients with renovascular hypertension and usually decreases after angioplasty [2]. Unilateral RAS is reportedly associated with NS, diagnosed as FSGS in the contralateral kidney. The mechanisms underlying the development of secondary FSGS in patients with RAS and renovascular hypertension include glomerular hypertension and hyperfiltration, activation of the renin-angiotensin II-aldosterone system by a high angiotensin II level, and injury of glomerular endothelial cells [6, 8]. Proteinuria dramatically improves following medical therapy or revascularization in most cases. However, Bhardwaj et al. suggested that immune-mediated injury, not a hemodynamic factor, is involved in development of this condition and reported that NS resolves after steroid therapy [13].

By contrast, reports have described increasing proteinuria after revascularization in patients with RAS. We identified three cases with NS after angioplasty. A literature search identified two patients similar to ours with FSGS in the ipsilateral kidney following revascularization of a single functioning kidney [11, 12]. These patients developed nephrotic-range proteinuria 2–4 weeks after angioplasty and showed hyperreninemia. One patient refused additional workup, and the other showed increased proteinuria after ACE inhibitor treatment was terminated due to renal function deterioration. Both patients died within 1 year. But, NS in our patient was resolved and he maintains stable CKD stage G3aA2 after renal angioplasty and treatment with an ARB for 1 year (Table 1). Another patient was diagnosed with RAS due to fibromuscular dysplasia and developed nephrotic-range proteinuria 2 days after angioplasty; however, NS spontaneously resolved within 1 week without any treatment [14].

**Table 1:** Clinical manifestations in three patients with FSGS after renal angioplasty

	Kanagasundaram et al. [11]	Almirall et al. [12]	This patient
Age (years)	65	72	48
Sex	Female	Male	Male
Blood pressure (mmHg)	220/120	240/120	160/90
Anti-HTN drugs	Amlodipine, doxazocin, furosemide	Amlodipine, lisinopril, hydrochlorothiazide	Carvedilol, hydrochlorothiazide
Serum creatinine (mg/dl)	2.07	1.90	1.42
Proteinuria before angioplasty	Normal	100 mg/day	0.4 g/g
Renal angiography			
Degree of RAS (%)	95	80	80
Opposite kidney	Non-perfused	Complete occlusion	Non-functional
Onset of nephrotic syndrome after angioplasty (weeks)	2	3	4
Proteinuria after angioplasty	5.9 g/day	13 g/day	11.6 g/g
PRA before angioplasty	–	–	19.32 ng/ml/hr
PRA after angioplasty (reference)	11.6 pmol/ml/hr. (2.8–4.5)	669 pg/ml (< 300)	60.6 mg/ml/hr. (1.31–3.95)
Management	ACEI		ARB
Change in proteinuria	3.1 g/day (2 weeks with ACEI)	16 g/day	2.2 g/g (9 weeks with ARB)
Follow-up	Proteinuria 6.5 g/day (after ACEI withdrawal)	Death due to ICH at 4 months after angioplasty	uPCR 0.4 g/g (1 year with ARB)

Abbreviations: *ACEI* angiotensin-converting enzyme inhibitor, *ARB* angiotensin receptor blocker, *HTN* hypertension, *ICH* intracranial hemorrhage, *PRA* plasma renin activity, *RAS* renal artery stenosis, *uPCR* urine protein-to-creatinine ratio

Several hypotheses may account for FSGS after angioplasty in this patient. First, in the presence of RAS, an ischemic change may be induced from the renal artery stenosis and the number of functional nephrons may be reduced, with hyperfiltration of the remnant functional nephrons induced by hemodynamic changes causing secondary FSGS without proteinuria for a long time before angioplasy [3, 15, 16]. Alternatively, a timely restoration of blood flow may be essential to salvage the ischemic renal injury. However, abrupt hyperfiltration injury due to reperfusion after angioplasty can paradoxically cause proteinuria via the damage to podocytes in the remnant ischemic tissue [17]. On the other hand, RAS can protect some glomerular injury. Bonsib et al. showed asymmetrical glomerular injury in a patient with unilateral RAS and glomerulonephritis. This protection from hyperfiltration and glomerular damage in the post-stenotic kidney can be removed by angioplasty and develop the nephrotic synrome [18]. These hemodynamic factors may cause FSGS because elevated intraglomerular hydrostatic pressure and over-distension of the glomerular capillary loop may injure podocytes.

The two-kidney one-clip (2K1C) model results in unilateral RAS. Perfusion is reduced in the stenotic kidney, and the renin-angiotensin system is activated, which stimulates the sympathetic nervous system and promotes hypertension. Perfusion is maintained in the contralateral non-stenotic kidney, and sodium and water are excreted via pressure natriuresis, with the overall plasma volume remaining normal. By contrast, the two-kidney two-clip (2K2C) model of bilateral RAS and the one-kidney one-clip (1K1C) model with a single functioning kidney, both of which lack a normal contralateral kidney, experience sodium retention, with the renin concentration being normal or somewhat reduced [19, 20]. The findings in our patient are compatible with the 2K2C model, with PRA increasing after angioplasty. Proteinuria resolved following ACE inhibitor treatment. The patient’s persistent hyperreninemia and response to renin-angiotensin-aldosterone system inhibition indicate that a hemodynamic factor was involved in proteinuria. His hyperreninemia may be related to renin production by remnant tissue of the non-functioning contralateral kidney.

Massive proteinuria after successful angioplasty is indicative of acute changes in hemodynamic factors. RAS may have been present in a single kidney, resulting in subclinical FSGS. Acute hyperfiltration upon reperfusion after angioplasty may have induced release of pro-inflammatory cytokines and oxidative stress from activated the renin-angiotensin-aldosterone system, leading to NS. These effects may be controlled by blocking the renin-angiotensin system, suggesting the need for periodic clinical follow-up. Large hemodynamic changes over a short period of time before and after angioplasty were speculated to be the cause of FSGS, although we report here development of proteinuria secondary to FSGS post revascularization, we can only speculate regarding the precise mechanism and this will need further investigation.

This report describes a patient with a single functional kidney and RAS who developed FSGS after renal angioplasty. Proteinuria in this patient was resolved following treatment with an ARB for 1 year. Changes in proteinuria should be evaluated in patients with renovascular hypertension before and after angioplasty. These patients may benefit from treatment with renin-angiotensin-aldosterone system inhibitors.

## Data Availability

The relevant clinical details are presented in this report.

## Abbreviations

ARB: Angiotensin receptor blocker
eGFR: Estimated glomerular filtration rate
FSGS: Focal segmental glomerulosclerosis
NS: Nephrotic syndrome
PRA: Plasma renin activity
RAS: Renal artery stenosis
sCr: Serum creatinine
uPCR: Urine protein-to-creatinine ratio
